# An integrated feature ranking and selection framework for ADHD characterization

**DOI:** 10.1007/s40708-016-0047-1

**Published:** 2016-04-02

**Authors:** Cao Xiao, Jesse Bledsoe, Shouyi Wang, Wanpracha Art Chaovalitwongse, Sonya Mehta, Margaret Semrud-Clikeman, Thomas Grabowski

**Affiliations:** 1 University of Washington, Seattle, WA USA; 2 University of Texas, Arlington, Arlington, USA; 3 University of Minnesota, Minneapolis, USA

## Abstract

Today, diagnosis of attention deficit hyperactivity disorder (ADHD) still primarily relies on a series of subjective evaluations that highly rely on a doctor’s experiences and intuitions from diagnostic interviews and observed behavior measures. An accurate and objective diagnosis of ADHD is still a challenge and leaves much to be desired. Many children and adults are inappropriately labeled with ADHD conditions, whereas many are left undiagnosed and untreated. Recent advances in neuroimaging studies have enabled us to search for both structural (e.g., cortical thickness, brain volume) and functional (functional connectivity) abnormalities that can potentially be used as new biomarkers of ADHD. However, structural and functional characteristics of neuroimaging data, especially magnetic resonance imaging (MRI), usually generate a large number of features. With a limited sample size, traditional machine learning techniques can be problematic to discover the true characteristic features of ADHD due to the significant issues of overfitting, computational burden, and interpretability of the model. There is an urgent need of efficient approaches to identify meaningful discriminative variables from a higher dimensional feature space when sample size is small compared with the number of features. To tackle this problem, this paper proposes a novel integrated feature ranking and selection framework that utilizes normalized brain cortical thickness features extracted from MRI data to discriminate ADHD subjects against healthy controls. The proposed framework combines information theoretic criteria and the least absolute shrinkage and selection operator (Lasso) method into a two-step feature selection process which is capable of selecting a sparse model while preserving the most informative features. The experimental results showed that the proposed framework generated the highest/comparable ADHD prediction accuracy compared with the state-of-the-art feature selection approaches with minimum number of features in the final model. The selected regions of interest in our model were consistent with recent brain–behavior studies of ADHD development, and thus confirmed the validity of the selected features by the proposed approach.

## Introduction

Attention deficit hyperactivity disorder (ADHD) is among the most common child and adult neurodevelopmental disorder. ADHD symptoms include inattention, hyperactivity, and impulsivity. It affects approximately 5–10 % of all school-age children and nearly 5 % of adults on their motor, cognitive, and emotional development [1]. Diagnosis of ADHD still remains a challenge, requiring long-term and extended involvement from clinicians, parents, and teachers. Clinicians rely heavily on experiences and intuitions when conducting diagnostic interview and observational measures. A delay or incorrect diagnosis of ADHD could have a significant negative impact on a patient’s social and emotional development, while an early and accurate detection of ADHD can strongly influence the course of the condition development by delivery of appropriate treatments to the patient. In addition to the traditional clinical diagnosis, there is a pressing need to find a set of more discriminative and objective features to characterize ADHD that can be used to facilitate ADHD diagnosis.

Previous studies on the etiology of ADHD are mostly based on structural or functional neuroimaging research of group level (ADHD vs. control) differences. Some informative features extracted are blood oxygenation level-dependent (BOLD) signals from functional magnetic resonance imaging (fMRI) data [2], wavelet synchronization likelihoods extracted from electroencephalography (EEG) data [3], rolandic spikes from EEG data [4], brain volume measure extracted from magnetic resonance imaging (MRI) data [5]. The pursuit of neuroanatomical biomarkers has a great potential to facilitate new discriminative methods that are etiologically informed and validated by neuropsychological theories. However, due to high cost of neuroimaging data acquisition, most current ADHD studies are based on relatively small sample sizes, which reduce the statistical power needed to validate meaningful discriminative variable from a very large number of features extracted from structural MRI [6]. A limited sample size with equivalent number of features raises new challenges to traditional machine learning algorithms, such as logistic regression or support vector machines (SVM), as they tend to overfit and lack a generalization power when training on a dataset containing the number of features far larger than the sample size ($p \gg n$ problem). In previous work, some models either use hundreds of features as an input or exhaustively search on a preselected smaller subset of features. SVM is mostly favored [7] and some variant of feedforward neural networks [8] is also used. We believe that those methods are either susceptible to overfitting or too restrictive in the search space. The interpretation of the final models is very difficult to validate by existing neuropsychological theories.

In this study, we propose an integrated feature ranking and selection framework that uses brain cortical thickness, extracted from structural MRI data, as features and constructs a prediction model to identify ADHD subjects versus normal controls. The framework performs a two-step feature selection process based on both information theoretic criteria and regularization concept. To mitigate the inconsistent feature selection issue of regularization, especially the lasso method [9], the framework preanalyzes all features to rank informative features based on mutual information scores [10]. In feature selection, it extends the lasso method [11] to construct a prediction model by fixing those preselected highly informative features when performing regression. Tested on both simulated and real datasets, our framework is shown to effectively preserve highly informative features identified in the feature ranking step and improve the model accuracy while searching in a full-feature space and maintaining the sparsity in the feature selection step. With a prediction accuracy of 80.9 %, our framework selects two sparse models, each with only 4 or 5 cortical thickness features. Previous neurodevelopmental studies of ADHD also consistently suggest that the features selected in our models have a deeper connection to the neurodevelopmental basis of ADHD, and thus making the models highly interpretable to clinicians. The proposed feature selection and prediction framework is a necessary first step to help clinicians find more features of characterizing ADHD using an objective measure with high discriminative accuracy.

The rest of the paper is organized as follows. In Sect. 2, we introduce the background of ADHD, including the brain cortical thickness and its connection to ADHD. We also review the current feature ranking and selection algorithms. Section 3 presents the proposed two-step feature ranking and selection framework, including the model formulations and model validation using simulated datasets. Section 4 shows the experimental results of the proposed framework on ADHD characterization using a real MRI neuroimaging dataset. Finally, we conclude the study in Sect. 5.

## Background

### Feature extraction of ADHD

ADHD is considered a neurodevelopmental disorder given the age-related differences in cortical maturation that characterize ADHD. Researchers suggest that the origins of attention can be observed in infants as young as three months when the young infant is able to selectively attend (i.e., recognize and orient toward) to their caregiver’s face [12]. According to these researchers, attention is composed of differential structures and circuits, called an organ system. Furthermore, as a child matures during preschool and early elementary school years, attention response grows into the ability to self-regulate (i.e., adjust one’s emotional state/behavior depending on the demands of the environment) in a changing and dynamic environment. Those higher level attention abilities are often described with the term “executive functions.” Such development not only relies on social demand, but also is due to the brain maturation of the prefrontal cortex. In Posner and Fan’s (2008) model, self-regulation leads to the second stage in attention development, the executive network. During the ages of 5–9, children with deficits in self-regulation and attention are noticed by teachers and parents, as their behaviors deviate from what would be developmentally appropriate.

Choosing brain cortical thickness as the features in ADHD characterization is not only supported by theory, but also benefits from advances of neuroimaging techniques. Numerous theories have hypothesized the cause of ADHD [13–18]. Those hypothesis are further supported by neuroimaging research, which provides an accurate way to measure the relationship between behaviors or symptoms and underlying brain morphology and brain functioning. As structural and functional neuroimaging techniques have improved vastly over the last thirty years, MRI provides excellent spatial resolution, uses no ionizing radiation (unlike computed tomography, CT), and thus can be used in pediatric samples of clinical and non-clinical typically developing controls. Cortical and surfaced-based neuroimaging techniques improve on conventional volumetric analysis by allowing for a direct measure of cortical thickness in millimeters, thus may present a more sensitive tool for understanding and measuring brain abnormalities in children with ADHD. So far, a large number of neuroimaging studies have observed that ADHD manifests via a general deficit in the dopaminergic system of the brain including prefrontal cortex [13, 5] or abnormalities in brain structures rich in dopamine receptors in children and adults with ADHD [19–23, 5].

### Feature selection

Although recent advances in neuroimaging studies have enabled us to search for structural brain abnormalities caused by the disease that can potentially be used as new biomarkers of ADHD, characterization using traditional machine learning techniques can be difficult because structural characteristics of neuroimaging data, especially MRI data, usually result in large number of features. Even grouping raw features into region of interests (ROI), finding discriminative features for ADHD is still not easy due to relative small sample size with a limited number of patients and healthy participants. Learning from limited sample size with equivalent feature size raises significant issues of overfitting and interpretability of the final model. This study is motivated by the challenge and is aimed to develop efficient feature selection approaches that can construct a sparse model with the most clinical meaningful features preserved. In particular, this paper proposes a novel integrated feature ranking and selection framework which combines information theoretic criteria and the least absolute shrinkage and selection operator (Lasso) method into a two-step feature selection process. The current information theory-based and the Lasso-based feature selection approaches will be discussed in the following.

#### Feature selection using mutual information

Mutual information [24, 10] is a measure of the inherent dependence expressed in the joint distribution of X and Y relative to the joint distribution of X and Y under the assumption of independence. MI measures how much information a feature contains about the class without making any assumptions about the nature of their underlying relationships. It is formulated as

$$I(X,Y)=\sum \limits _{y\in Y} \sum \limits _{x\in X}p(x,y) \log \left( \dfrac{p(x,y)}{p(x) p(y)} \right) .$$

If the feature is a perfect indicator for the class membership, its MI reaches its maximum value. A basic intuition is that a stronger mutual information implies a greater predictive ability when using the feature. As an information theoretic criteria, MI have been applied in many feature selection problems [25]. To know whether a given candidate feature should be included, one must be able to evaluate the joint mutual information *I*(*X*, *Y*). However, as feature matrix *X* is generally multi-dimensional with a continuous distribution, the joint mutual information *I*(*X*, *Y*) is thus extremely difficult to reliably estimate. To solve the problem, one can assume each feature is independent of all other features, and rank the features in descending order according to their individual mutual information score $I(X_i, Y )$. The feature selection is simply picking the top *K* features, where *K* can be determined by a predefined certain number of features or some stopping criterion. The feature selection criterion based on mutual information score is commonly adopted in literature. It is often referred as Mutual Information Maximization (MIM) approaches [26]. However, the performance of this approach is known to be suboptimal if features are interdependent, which is a general case in most studies. In addition, it is widely accepted that a useful set of features should not only be individually relevant to class label, but also should not be redundant with respect to each other, that is features should not be highly correlated in the selected subset. To consider both relevancy and redundancy, a number of approaches have been proposed. For example, Battiti [27] proposed the Mutual Information Feature Selection (MIFS) criterion, which introduces an inter-feature correlation term into the MIM criterion. A penalty parameter $\beta$ is employed to control the tradeoff between relevancy and redundancy. If the penalty parameter $\beta$ is set to 0, it is equivalent to the MIM criterion. Peng et. al. [28] presented the Maximum-Relevance-Minimum-Redundancy (MRMR) criterion, which is in principle equivalent to MIFS with the $\beta = 1/(n-1)$, where *n* is the number of selected features in the current subset. Yang and Moody [29] used Joint Mutual Information (JMI) to focus on increasing complementary information between features. In particular, the mutual information between the class label and a joint random variable $X_kX_j$ is calculated. By pairing a candidate $X_k$ with each previously selected feature. The principle idea is that if the candidate feature is ‘complementary’ with the existing features, it should be included in the feature subset. Fleuret proposed the Conditional Mutual Information Maximization (CMIM) criterion [30], which examines the information between a feature and the class label, conditioned on each current feature. Instead of taking the mean of the redundancy term, CMIM takes the maximum value in the redundancy term and thus penalize more on feature redundancy.

Although mutual information-based feature selection approaches gained wide popularity in the literature, there are still some significant issues unsolved. First, all these criteria rely on highly restrictive assumptions on the underlying data distributions. In particular, due to the computational difficulties in high-dimensional mutual information estimation, most approaches only consider pairwise and conditional pairwise interactions, and omit the higher-order interactions. Second, most current MI-based approaches perform feature selection sequentially starting from high-ranked features. As a result, by excluding low MI ranking features, such approaches deny the possibility that a set of low-ranked features combined together may generate strong predictive power (e.g., in the famous XOR problem [31]). We have the risk of missing that strong signal by only working on the preselected candidate set [32, 33, 28].

#### Feature selection with regularization

In medical research, due to high cost of data acquisition, researchers often run into the issue of insufficient samples to train and validate developed models. Instead of heuristic selection schemes (such as many MI-based approaches), objective optimization methods have received more attention since they can be conveniently formulated as convex optimization problems with global optimal solutions. A typical objective function consists of an error term and a regularization term. One of the most widely used such feature selection algorithms is the least absolute shrinkage and selection operator (Lasso) [34]), which allows computationally efficient feature selection based on linear dependencies between input features and output values. The Lasso method as a shrinkage and selection method for linear regression gradually receives high recognition and a fast coordinate descent algorithm has been devised to solve the optimization problem. The optimization framework of lasso to minimize the sum of squared errors with a $l_1$-norm penalty (bound on the sum of the absolute values of the coefficients) is formulated as follows:

$$\sum \limits _{i=1}^{n}\left( y_i - \beta x_i \right) ^2+\lambda ||\beta ||_1.$$

By penalizing and forcing some variables to be zero, lasso can effectively select a sparse model. However, it sacrifices unbiasedness to reduce the variance of the predicted value [35].

There are still some challenges for application of Lasso method in feature selection. The Lasso result is often subject to the scaling of features. Inappropriate scaling may cause imbalanced penalty on linear coefficients. The true underlining features with high coefficients may be suppressed to have smaller coefficients. As a result, the total explained variance is limited. Instead of rescaling all features, more generally one can employ adaptive Lasso [36] with penalty term $\lambda \sum w_i||\beta _i||_1$. Even so, effects of strong signal will be diminished due to shrinkage.

## New integrated feature ranking and selection model

### Model formulation and solution

**Fig. 1:**
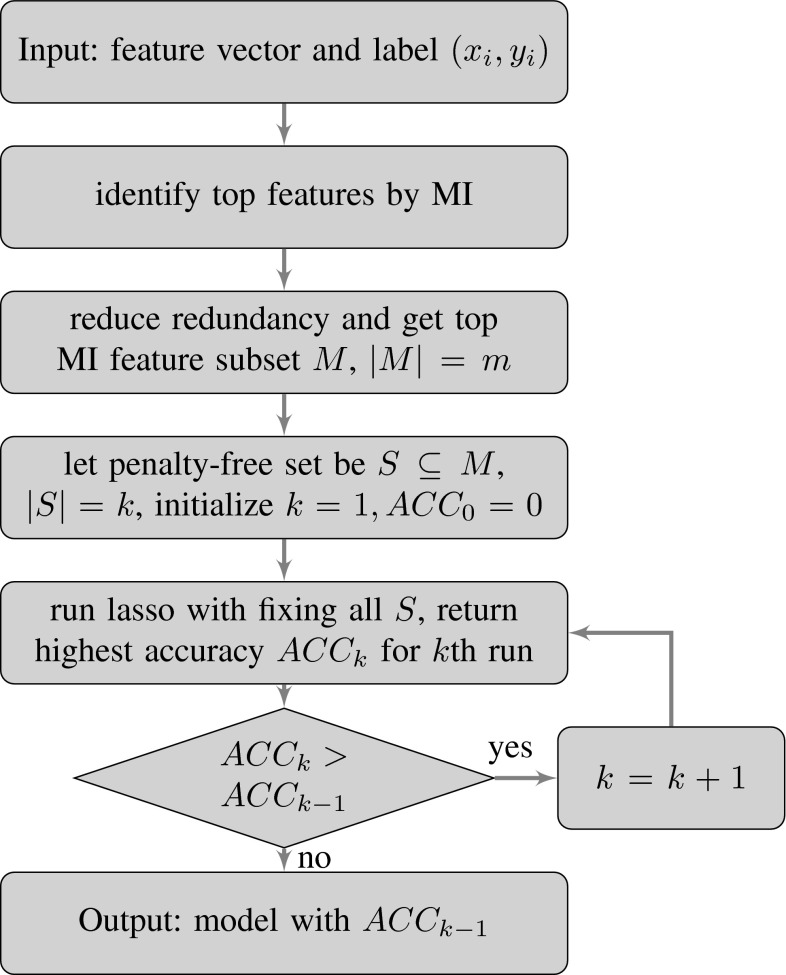
Flowchart of Integrated Feature Ranking and Selection Model

The proposed integrated feature ranking and selection framework is performed in two stages: mutual information-based feature ranking and Lasso-based feature selection. In the feature ranking step, all features are ranked by their MI scores, and a subset of high-ranked features are selected and considered to have the best informative power. Among those features, a redundancy removal step is performed by checking pairwise correlation between the features. For a highly correlated feature pair (higher than a threshold), the feature with lower MI score is considered redundant and removed from the feature subset to prevent multicollinearity. In the feature selection step, we set the best informative features penalty-free in the generalized lasso method. We use Lasso to select additional features from the full-feature space, not restricted to the subset of high MI features. The additional features selected, although have lower MI scores individually, can improve model classification performance when combined together. Within the subset of high MI features, we start with setting the single top-ranked feature penalty-free, then all combinations of two top features, then all combinations of three top features, iteratively. The feature selection and classification model was validated by leave-one-out cross-validation (LOOCV). The search process stops when validation accuracy cannot be further improved. The resulting model will be the best model for class prediction. Comparing with other MI-ranking-based methods, the proposed framework can select from the full-feature space while still creating a sparse model. Comparing with standard regression approaches with regularization, the proposed framework integrates the information theoretic criteria in the generalized Lasso model, and sets the most informative features penalty-free to improve prediction accuracy and enhance model interpretability. The flowchart of the proposed integrated feature ranking and selection framework is shown in Fig. 1.

Mathematically, our framework can be formulated as an optimization problem. Let *M* be the set of indexes of top MI features selected from the MI ranking step. We set indexes in *S* penalty-free, where *S* is a subset of *M*. For each *S*, we want to solve the following problem.

1 $$\begin{aligned}&\underset{\beta }{\min }&\sum \limits _{i=1}^{n}\bigg (y_i-\beta _0-\sum \limits _{j=1}^{p}\beta _jx_{ij} \bigg )^2 + \lambda \sum \limits _{j=1}^{p}|\beta _j| \end{aligned}$$

2 $$\begin{aligned}{\text{ s.t. }}&\beta _{j\in S}=0.\end{aligned}$$

The optimization model in our Integrated Feature Ranking and Selection Framework can be solved under generalized lasso framework [37], which is more flexible than lasso and is better in representing the intention to set coefficients of certain informative features penalty-free. Basically, it introduces an arbitrary matrix $D\in {\mathbb {R}}^{m\times p}, m\le p$ to define the weights and relations of each element in $\beta$.

$$\underset{\beta \in {\mathbb {R}}^p}{\min }\qquad \big |\big | y- X\beta \big |\big |_2^2 + \lambda \big |\big |D\beta \big |\big |_1.$$

We can construct a proper *D* in the generalized lasso framework to adjust penalty levels for each feature. To find such a *D*, we propose and prove the following two propositions.

#### **Proposition 1**

$$\underset{\beta \in {\mathbb {R}}^p}{\min } \ \big |\big | y- X\beta \big |\big |_2^2 + \big |\big |\big (\lambda _1\beta _1, \lambda _2\beta _2, \ldots , \lambda _p\beta _p \big )\big |\big |_1.$$

The above problem of assigning weights $\lambda _k$ for each feature is equivalent to the generalized lasso with diagonal matrix *D* and $\lambda _k = d_k \lambda$. (The above formula has also been previously presented as adaptive lasso [36].)

#### *Proof*

Let D be diagonal matrix ${\text {diag}}(d_1, d_2, \ldots , d_p)$, we have

$$\lambda \big |\big |D\beta ||_1 = \lambda \big |\big | \big (d_1 \beta _1, d_2 \beta _2, \ldots , d_p \beta _p\big )^T \big |\big |_1 = \big |\big |\big (\lambda _1\beta _1, \ldots , \lambda _p\beta _p \big )\big |\big |_1.$$

If D is $p\times p$ and invertible, $\beta$ can be transformed into $\theta = D\beta$. The generalized form can be reduced to the standard lasso:

$$\underset{\theta \in {\mathbb {R}}^p}{\min }\qquad \big |\big | y- XD^{-1}\theta \big |\big |_2^2 + \lambda \big |\big |\theta \big |\big |_1.$$

$$\square$$

#### **Proposition 2**

Without loss of generality, to keeping features $X_{p-k+1}, X_{p-k+2}, \ldots , X_{p}$ penalty-free is equivalent to setting $d_{p-k+1} = 0, d_{p-k+2} = 0, \ldots , d_{p} = 0$.

#### *Proof*

In this case, D is a rank-deficient matrix

$${\text {diag}}(d_1, d_2, \ldots , d_{p-k}, 0, \ldots , 0).$$

$$\lambda \big |\big |D\beta \big |\big |_1 = \big |\big |\big (\lambda _1\beta _1, \lambda _2\beta _2, \ldots , \lambda _{p-k}\beta _{p-k} \big )\big |\big |_1.$$

Following the construction procedures in [37], we can transform and reduce the problem to a standard lasso problem. First, we create a full rank matrix $\tilde{D}$ by removing the last *k* rows from *D* and adding $k\times p$ matrix *A* to the bottom, where $m=p-k < p$.

$$\begin{aligned} \tilde{D}=\begin{bmatrix} d_1&\quad \cdots&\quad 0&\quad 0&\quad \cdots&\quad 0\\ \vdots&\quad \ddots&\quad \vdots&\quad&\quad&\quad \\ 0&\quad \cdots&\quad d_m&\quad&\quad&\quad \vdots \\ 0&\quad&\quad&\quad 1&\quad \cdots&\quad 0\\ \vdots&\quad&\quad&\quad \vdots&\quad \ddots&\quad \vdots \\ 0&\quad&\quad \cdots&\quad 0&\quad \cdots&\quad 1 \end{bmatrix}_{p\times p} \end{aligned}.$$

In the above matrix $\tilde{D}$, *A*’s rows are clearly orthogonal to those in *D*. Let $\theta =\tilde{D}\beta =(\theta _a, \beta _b)^T$, where $\theta _a$ is related to the coefficient vector $\beta _a$ of the first *m* features that are not in the desired set. Now the objective function is

$$\underset{\theta \in {\mathbb {R}}^p}{\min } \ \big |\big | y- X_a\theta _a - X_b\beta _b\big |\big |_2^2 + \lambda \big |\big |\theta _a\big |\big |_1,$$

where $X_a$ is the rescaled first *m* columns of *X*, $X_b$ is the original last *k* columns.

We optimize $\beta _b$, $\theta _a$ in a sequential way. First, fixing $\theta _a$, the problem regarding $\beta _b$ is a standard linear regression. The new objective function is to

$$\underset{\theta _a\in {\mathbb {R}}^m}{\min } \ \big |\big | (1-P)y- (1-P)X_a\theta _a\big |\big |_2^2 + \lambda \big |\big |\theta _a\big |\big |_1,$$

where $P=X_b(X_b^TX_b)^{-1}X_b^T$. We get a standard lasso problem regarding $\theta _a$. After solving $\theta _a$, we can in turn determine $\beta _b$ by $\hat{\beta _b} = (X_b^TX_b)^{-1}X_b^T(y-X_a\hat{\theta _a})$ from the result of linear regression. The solution of the original generalized lasso solution is $$\hat{\beta } = \tilde{D}^{-1}\hat{\theta } = \tilde{D}^{-1}\begin{bmatrix} \hat{\theta _a}, \hat{\beta _b} \end{bmatrix}^T$$. $\square$

Despite the formulation similarity between our model and adaptive lasso [36], adaptive lasso was previously proposed to include a data-dependent weight vector *w*. The weight vector is estimated as $\hat{w} = 1/|\hat{\beta }|^\gamma$ and no element is intended to be zero. From the formulation perspective, adaptive lasso is a special case of generalized lasso with a full-rank diagonal matrix. In our case, we construct *D* as a (0,1)-matrix that has exact one non-zero element in each row (i.e., $\sum _j d_{ij}=1$) and at most one non-zero element in each column (i.e., $\sum _i d_{ij}\le 1$). The column indices of non-zero elements are the features subject to $l_1$ penalty. The complement set of $p-m$ features are those, we believe, that are information rich and thus set penalty-free.

### Performance evaluation using simulated dataset

**Fig. 2:**
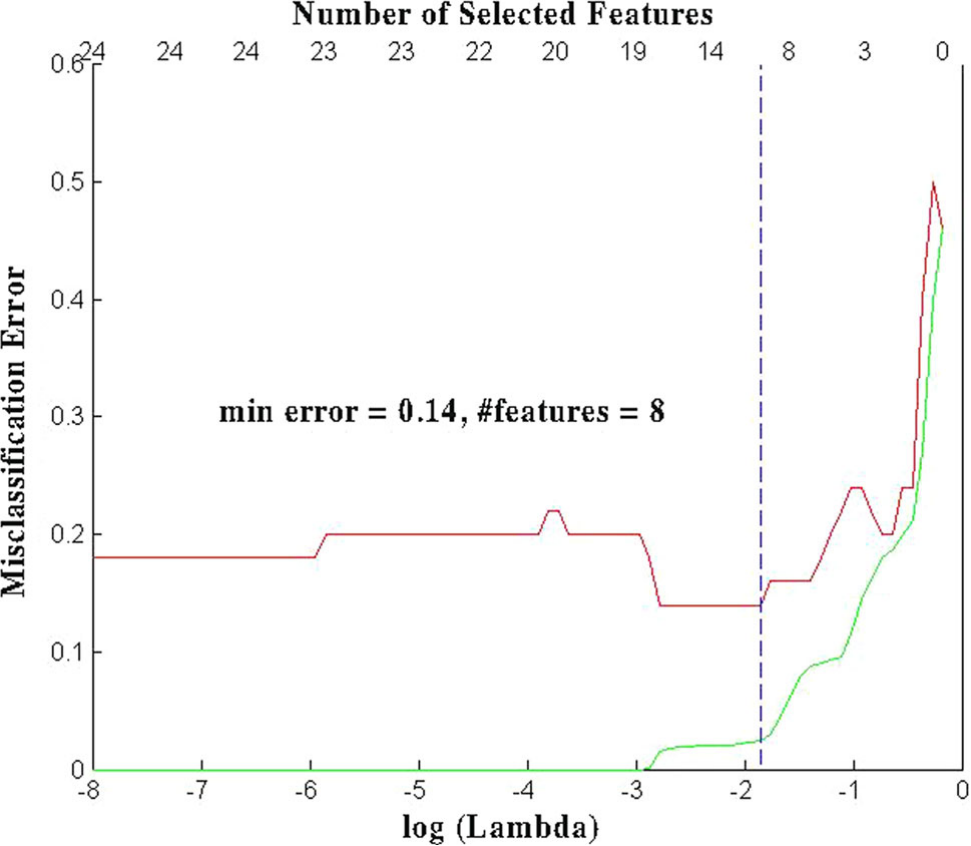
Best prediction error using LR + lasso (*green curve* as training error, *red curve* as testing error, *dashed line* cuts at min testing error)

**Fig. 3:**
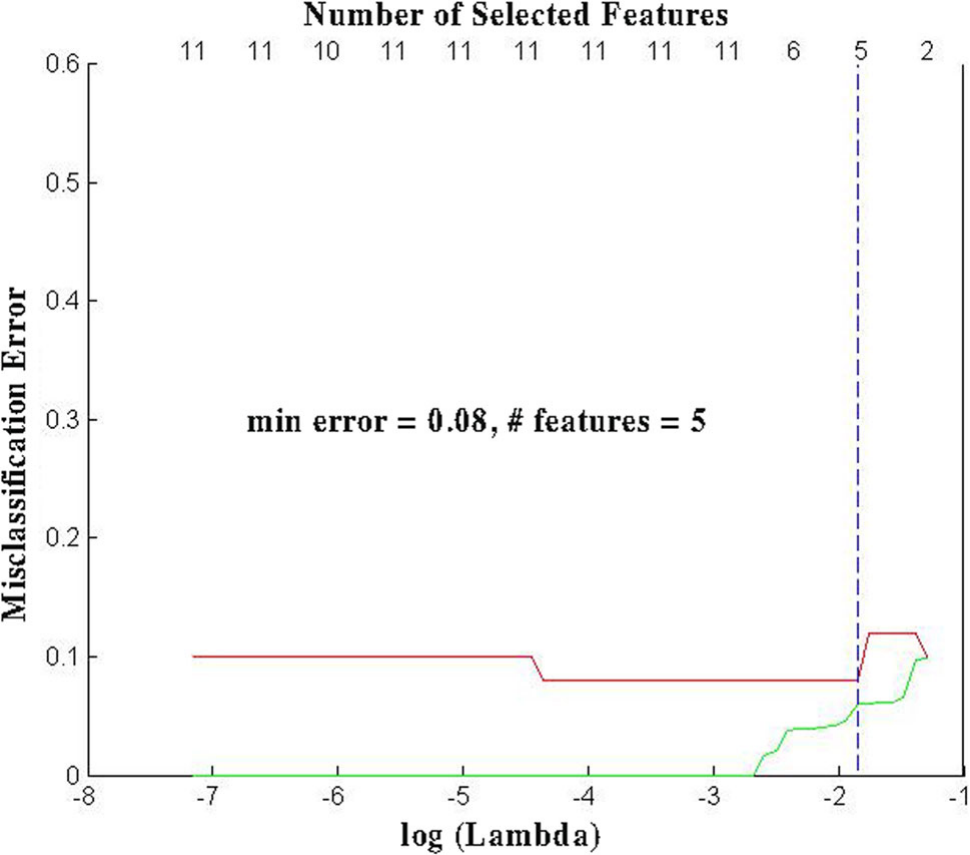
Best prediction error using our framework (*green curve* as training error, *red curve* as testing error, *dashed line* cuts at min testing error)

To evaluate the performance of the proposed feature selection framework, we used a simulated dataset with binary response and contain $p=45$ predictors and $n=50$ samples. The dataset was generated in such a way that only two predictors were related to the response. Using LOOCV, the proposed framework achieved a validation accuracy of 0.92 with five features selected. As a comparison, we also tested the logistic regression (LR) with lasso, which generated a validation accuracy of 0.86 with 8 features selected. The detailed comparison results are summarized in Table 1 as well as Figs. 1 and 2. From those results, one can see clearly that the proposed framework is capable of selecting a model with higher validation accuracy while with less selected features compared to lasso (Fig. 3).

**Table 1:** Performance comparison on simulated dataset

Method	Validation accuracy	Training accuracy	Features selected
Our Model	0.92	0.94	5
LR + lasso	0.86	0.97	8

## Application in the diagnosis of ADHD

### Dataset

This study used a dataset that was collected as part of a larger study from the University of Texas at Austin and the University of Texas Health Science Center in San Antonio by Dr. Margaret Semrud-Clikeman.

A total of 47 subjects matched on gender, SES, and ethnicity participated in the study. All subjects were right handed. There were two groups: 32 ADHD-Combined participants and 15 healthy subjects in a control group. All ADHD participants had less than 15 standard score point differences between general conceptual ability (DAS-GCA) and all achievement measures. The ADHD subjects were matched on severity of symptoms as measured by Conners’ Ratings Scale (Conners, 1998a). All ADHD subjects met DSM IV-TR criterion for ADHD Combined-type and no other psychiatric or psychological disorder including Learning Disorders, Anxiety Disorders, Mood Disorder, or Oppositional Defiant Disorder. Control participants did not meet any criteria for a psychiatric or learning diagnosis nor have a history of medication treatment. All participants were recruited from a diversity of socioeconomic and ethnic backgrounds in order to control for potential group differences.

MRI images are acquired at the University of Texas Health Science Center at San Antonio using three-dimensional gradient recalled acquisitions in the study state (3D-GRASS) with a repetition time (TR) = 33 msec, echo time (TE) = 12 msec, and a flip angle of 60 degrees to obtain a 256 $\times$ 192 $\times$ 192 volume of data with a spatial resolution of 1mm $\times$ 1mm $\times$ 1mm. Then all MRI images were processed and normalized using the FreeSurfer image analysis suite [38, 39] by Dr. Jesse Bledsoe on a Linux platform at MSU. All regions of interest (ROI) in the FreeSurfer suite (45 cortical ROIs) were developed using an automated labeling system based on gyral regions of the Desikan-Killiany Atlas [40]. We employed the brain cortical thickness of those ROIs as possible features for ADHD feature characterization and selection in this study.

### Results of feature ranking and decorrelation step

The first step in our framework is to perform feature ranking using mutual information. The top ten features of cortical thickness with highest MI were picked first for further analysis. They are right rostral anterior cingulate (MI = 0.124), total rostral anterior cingulate (0.122), left rostral anterior cingulate (0.078), left caudal middle frontal (0.071), right frontal pole (0.068), right lateral orbito frontal (0.063), left caudal anterior cingulate (0.062), total caudal middle frontal (0.051), left inferior parietal (0.051), and left pars orbitalis (0.05). In the next step, we calculated the correlation between each pair of the high-ranked 10 features. If the correlation of a pair of features is 0.6 or higher, we consider one feature in the pair to be redundant, and remove the feature with a lower mutual information value. In this way, the following two features were removed: total rostral anterior cingulate and total caudal middle frontal. The remaining eight features were used as feature candidates in the Lasso-based feature selection step.

### Results of feature selection step

#### Comparison of testing accuracy

In feature selection step, within top eight highest MI and uncorrelated feature set, we started with fixing the single top feature penalty-free, then all combinations of two features penalty-free, then all combinations of three features, iteratively. We evaluated the selection and prediction model using the validation accuracies in a LOOCV procedure. The model search process stops at fixing four features penalty-free, as when fixing more features, the validation accuracy started to decrease. The resulting model is the best prediction model with the highest LOOCV validation accuracy. As shown in Table 2, the proposed framework achieved a testing accuracy of 0.81 with a sensitivity of 0.81 and a specificity of 0.80.

In addition, we also tested and compared the performance of the state-of-the-art feature selection algorithms, including the aforementioned information theoretic methods MRMR [28], MIFS [27], JMI [29], CMIM [30], MIM [26], as well as the popular Pudil’s floating search method [41], and the principle component analysis (PCA)-based approach, for which we took the components that account for 95 % of data variance as the selected features in prediction. The prediction results of these approaches are also summarized in Table 2. One can observe that the proposed method achieved higher validation accuracy (0.81) than all other compared feature selection approaches, while using the lowest number of features in the final prediction model. These experimental results confirmed that our model is efficient to select the most predictive features of ADHD given a small sample size.

**Table 2:** Comparison of testing results (leave-one-out cross-validation)

Selected features	Testing accuracy	Training accuracy	Sensitivity	Specificity	Selection method
4	0.81	0.87	0.81	0.80	Proposed method
5	0.76	0.78	0.75	0.80	MRMR [28]
7	0.66	0.76	0.66	0.67	Pudil’s floating search [41]
14	0.70	0.74	0.72	0.67	PCA
5	0.74	0.75	0.81	0.60	MIM [33]
5	0.70	0.76	0.69	0.73	MIFS [27]
5	0.72	0.78	0.72	0.73	JMI [29]
5	0.74	0.76	0.75	0.73	CMIM [30]

#### Analysis of features in best models

To investigate the model interpretability, we also checked the locations of the selected cortical thickness. All the features (regions of interest) selected by the best models were located in prefrontal cortex (PFC), anterior cingulate cortex, and parietal cortex. Structural and functional impairments are in accordance with current understanding of brain–behavior relationships in ADHD.

The prefrontal cortex (PFC) is connected with nearly every cortical structure of the central nervous system [42] and is involved in nearly all aspects of human personality and cognition. The PFC has received much attention in the ADHD literature given a large body of research on impairments in tests thought to tap PFC functioning [43, 44]. For example, the PFC has been implicated in complex behavior relevant to central impairments in ADHD such as inhibitory control [45, 46], attention, working memory, and planning [42, 47]. Furthermore, specific differences within the frontal pole and orbital frontal cortex observed here may provide further evidence for impairments in frontal limbic structures and emotional disorders which often co-occur in children with ADHD [48].

The anterior cingulate cortex is a key structure implicated in attentional control [47]. It is implicated in a wide variety of cognitive operations including response inhibition, reward processing, behavioral motivation, target detection, and decision making [49]. Functional neuroimaging studies suggest hypoactivation of areas of the anterior cingulate in children and adults with ADHD [50–52]. Studies observed decreased activation of the anterior cingulate in tasks thought to require behavioral inhibition (e.g., counting Stroop task) in children with ADHD compared to controls [50, 52] also reported reduced activation of the anterior cingulate during tasks of behavioral inhibition (e.g., stop signal task) in children with ADHD-C. Further, cortical thinning of the anterior cingulate cortex has been demonstrated in adults with ADHD [53]. Moreover, the right rostral anterior cingulate cortex (ACC) contributed the most predictive variance in classifying those with ADHD from typically developing controls. This finding supports the hypothesis that abnormal development of the the right ACC, in particular, may be considered a biomarker for ADHD and inhibitory control [54]. The ACC is likely implicated in ADHD due to its involvement in complex behavior. However, the ACC, itself, is unlikely to contribute to impaired attention. Rather, future work will need to address the complex networks and systems that involve the ACC in order to provide valid causal pathways for ADHD.

The left inferior parietal cortex also contributed to the classification of ADHD versus healthy children. This was a particularly interesting finding given recent work that has implicated abnormalities in parietal cortex during resting-state functional MRI [55]. Prior to this work, the posterior cortex was proposed to underlie the basis for arousal and vigilance which were considered precursors for targeted attention [47, 56]. And, more recent work has found the posterior parietal lobe to be important for shifting attention during dynamic attention tasks [57]. Structurally, reduced cortical thinning of the right-parietal cortex has also been observed in adults with ADHD [53]. Taken together, the parietal cortex, likely due to its frontal projections, is another important area in the attention network that may undergo abnormal development in those with ADHD.

The prefrontal cortex, anterior cingulate cortex, and parietal cortex have all been implicated in attentional control and ADHD. Given these regions provided the best classification of ADHD from controls, the proposed model would appear to be theoretically valid. A significant advantage of the proposed approach is that we novelly integrate the information theoretic feature selection framework with the generalized lasso framework. Through adaptively manipulating penalty weights of each feature in regularization term, we are able to preserve the most informative features in the final model and eliminate less informative and redundant features.

## Conclusion

ADHD feature characterization and selection has never been an easy task. In this paper, the proposed integrated feature ranking and selection framework provides a sparse, accurate, and highly interpretable model to assist ADHD feature characterization. With the proposed two-step formulation, one can integrate information theory conveniently to supervise the feature selection process while the optimal solutions can be guaranteed due to the convex optimization formulations in a generalized lasso framework. The information-guided selection structure enforces the most useful discriminative predictors to be included in the final prediction model while eliminating less-informative and redundant variables to create an accurate sparse prediction model. In addition to mutual information, due to the flexible structure of the proposed framework, one can also conveniently integrate clinical prior knowledge into the feature selection model. For example, one can set clinician-identified potentially important features penalty-free and encourage them to be included in the final prediction model. The information theory-guided and clinical prior knowledge-guided feature selection framework will be greatly useful to construct prediction models that are more transparent and interpretable by medical and healthcare professionals. Such a supervised feature selection framework is highly demanded in making clinical decisions compared to the ‘black box’ predictive models generated by traditional machine learning algorithms. As this is a general feature selection approach, the proposed technique can also be applied to other decision-making problems that require interpretable prediction models. The research in this study also suggest that machine learning techniques can be useful tools for understanding and measuring brain abnormalities associated with ADHD.
